# Production of unstable proteins through the formation of stable core complexes

**DOI:** 10.1038/ncomms10932

**Published:** 2016-03-17

**Authors:** Nicolas Levy, Sylvia Eiler, Karine Pradeau-Aubreton, Benoit Maillot, François Stricher, Marc Ruff

**Affiliations:** 1 IGBMC, UDS, CNRS, INSERM, 1 rue Laurent Fries, 67404 Illkirch, France

## Abstract

Purification of proteins that participate in large transient complexes is impeded by low amounts, heterogeneity, instability and poor solubility. To circumvent these difficulties we set up a methodology that enables the production of stable complexes for structural and functional studies. This procedure is benchmarked and applied to two challenging protein families: the human steroid nuclear receptors (SNR) and the HIV-1 pre-integration complex. In the context of transcriptional regulation studies, we produce and characterize the ligand-binding domains of the glucocorticoid nuclear receptor and the oestrogen receptor beta in complex with a TIF2 (transcriptional intermediary factor 2) domain containing the three SNR-binding motifs. In the context of retroviral integration, we demonstrate the stabilization of the HIV-1 integrase by formation of complexes with partner proteins and DNA. This procedure provides a powerful research tool for structural and functional studies of proteins participating in non-covalent macromolecular complexes.

## Abstract (web summary)

Flexible or disordered domains often hinder the purification of proteins involved in functional interactions. Here the authors describe an approach that enables the production of stable and functional complexes of otherwise unstable proteins in quantities sufficient for structural and functional studies.

## Graphical abstract

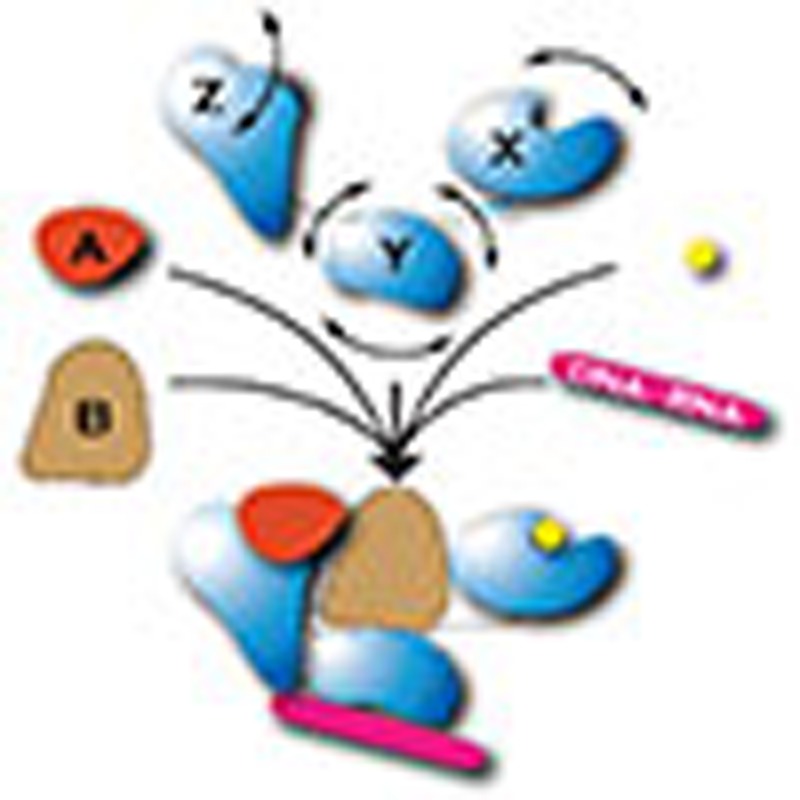

Protein flexibility and disorder have been shown to be inherent properties of major protein families^1^,^2^ involved in large transient non-covalent complexes^3^,^4^. This intrinsic disorder is often considered an evolutionary asset that allows proteins to interact with multiple partners to ensure multiple functions^5^,^6^ by mechanisms such as coupled folding and binding or conformational selection^7^. One consequence is that production and purification of such proteins are impeded by low amounts, heterogeneity, instability and poor solubility. Here we present a new methodology that enables the production of stable and functional complexes of proteins and/or protein domains in large amounts. It is based on the concept that each function of a protein with multiple activities corresponds to a unique structure stabilized and solubilized by the interaction with partner molecules^8^,^9^,^10^. This methodology has led to important biological results with two challenging protein families, namely the human steroid nuclear receptors (SNRs) and the HIV-1 pre-integration complex described in this and previous publications^11^,^12^,^13^,^14^.

To produce and purify proteins participating in these transient macromolecular complexes we set up a pipeline procedure to reconstitute complexes *in vitro* or *in cellulo* by assembling the proteins around the central core protein player of the complex (Fig. 1). Following this method, we demonstrate that the instability and poor solubility of two important protein families, the SNRs participating in human transcription activation complexes and the retroviral integrase participating in the HIV-1 pre-integration complex, could be overcome by forming long-lasting, stable, specific complexes with their ligands, DNA substrates or co-factor proteins. The general strategy enables the efficient screening of several parameters (core and partner protein sequences, solubilizing and purification tags, expression conditions, expression organism, solubilizing and stabilization buffer) leading to the production of stable complexes.

In the context of transcriptional regulation studies, we are able to produce and purify stable complexes between the oestrogen receptor beta and the glucocorticoid receptor (GR) with a full domain of the transcriptional intermediary factor 2 (TIF2) co-activator containing the three nuclear receptor-binding motifs. The latter is the first stable complex of GR with a full domain of a partner protein. We demonstrate that one molecule of TIF2 is bound to a dimer of the SNR and that TIF2 binds to SNRs through an induced folding mechanism. In the context of studies on the mechanism of retroviral integration, we produce the HIV-1 IN/LEDGF lens epithelium-derived growth factor complex in *E. coli*, insect and mammalian cells. We demonstrate here that an increase in solubility and activity is achieved using mammalian cells for the production and reconstitution of this complex.

## Results

### General strategy

**Figure 2:**
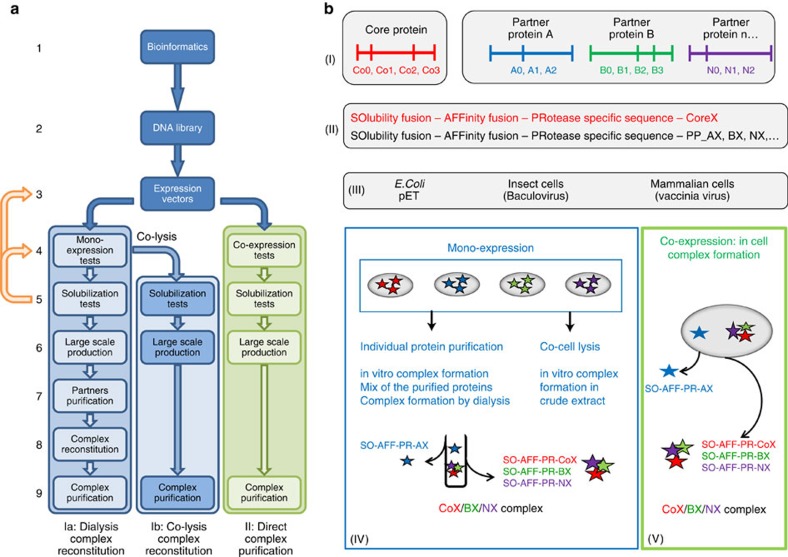
General strategy used for production of stable complexes. (**a**) Flowchart describing the 9 steps used in the pipeline. After bioinformatics analysis (**a1**) to define protein domains (**bI**), a DNA library is created. The proteins are then cloned in fusion with solubility and affinity tags (**bII**). Next they are transferred into expression vectors (**a3**) for protein production in prokaryotic and eukaryotic cells (**bIII**). Two strategies are then tested. One is the production of each protein individually **(a4**,**bIV**) and complex reconstitution by dialysis (**a**, line **Ia**) or co-cell lysis (**a**, line **Ib**) and another is the co-expression of the different proteins together (**a**, line II; **bV**). After solubility tests, steps 3–5 are recycled until the optimal conditions for the proteins/complex solubility and stability are found. After a large scale production (**a6**) protein purification (**a7**, line **Ia**) and complex reconstitution (**a8**, line **Ia**) the complex is purified by affinity purification and size exclusion chromatography.

The general strategy for the isolation of stable complexes follows a nine-step flowchart (Fig. 2). Analysis of protein sequences, the first step of our strategy, begins with the realization of multiple sequences alignments. The assumption is made that sequence homologies reveal conserved domains most often of functional and/or structural significance. Sequence alignments were performed using the PipeAlign^15^ software. Coupled with the multiple alignments, disorder analysis (PONDR-FIT^16^ and IUPRED^17^), domain prediction (CDD^18^), solubility predictions (SOLpro in SCRATCH^19^ programme suite and PROSO^20^) and secondary structures predictions (JPRED^21^) were performed. The analysis of the predictions of secondary and intrinsically disordered regions was used to define the boundaries of structural and functional domains with the hypothesis that a predicted domain is more likely to be soluble when its boundaries do not interrupt secondary structures; hydrophobic sequence stretches or intrinsically disordered regions. The delineation of the precise ends of structured or disordered domains was carried out through the joint use of these different analyses. When structures and/or homologous structures are known, structures and/or three-dimensional homology modelling using MODELLER^22^ through the CHIMERA^23^ interface as well as COOT^24^ through the CCP4 (ref. 25) interface helped to define the limits of structural domains. In case of high-protein instability, surface cysteine and/or hydrophobic residues were mutated.

In a second phase, these domains were used in parallel with the full-length genes to implement a DNA library. The resulting cDNAs were combined in frame to a set of DNA sequences encoding domains known to improve protein solubility as well as to affinity purification tags and protease recognition sequences that enable removal of the tags in a final step.

Starting from the third step, two sub-strategies were tested in parallel (I and II in Fig. 2). The first involves the production and purification of individual partners that were used for *in vitro* complex reconstitution by dialysis (Fig. 2a, line Ia) or by co-cell disruption (Fig. 2a, line Ib). The second involved the purification of the complex directly from cells (Fig. 2a, line II). For this purpose, the gateway technology was used to transfer the complementary DNAs (cDNAs) to expression vectors for single expression or co-expression, in bacterial, insect or mammalian cells. Different expression conditions were tested in small culture volumes by the variation of the composition of the culture medium and the temperature of induction. This step sometimes involved the addition of specific ligands during expression to ensure efficient production of soluble proteins/complexes.

Each successful expression test was followed by the optimization of solubilizing conditions. Cells were broken and extracts were clarified by centrifugation. Soluble and insoluble fractions were loaded on SDS–polyacrylamide gel electrophoresis (PAGE) to assess the solubility in the tested buffer. Different compositions (variation of pH, ionic force, presence and nature of detergent, and stabilizing agents) of lysis buffer were tested to optimize the solubility and stability of the individual proteins or complexes. Several iterative rounds from step 3 to step 5 (Fig. 2a) could be required to find the best combination of conditions.

Once optimal conditions were identified, large scale production was performed (step 6 in Fig. 2a). In the case of single-protein expression, partners are purified in two steps (affinity and size exclusion chromatography) and all purification tags removed except for one which was retained to allow final purification of the complex. The complex is then reconstituted by mixing the partners before the removal of the solubilizing agent through dialysis. Reconstituted complexes were finally purified in two steps (affinity and size exclusion chromatography). This method has been benchmarked with two challenging protein families, the human SNRs and the HIV-1 pre-integration complexes (PICs).

### Steroid nuclear receptors

Nuclear receptors are ligand-dependent transcription factors that play an important role in a variety of biological processes, including cell proliferation, differentiation and cellular homoeostasis^26^. They act in cell-type and gene-specific manners and regulate numerous physiological and pathological processes. Nuclear receptors typically contain about 600 amino-acid residues and have a modular domain structure consisting of a highly variable amino-terminal domain, a conserved central DNA-binding domain followed by the hinge domain, the ligand-binding domain (LBD) and the carboxy-terminal domain which has variable sequence (Supplementary Fig. 1)^27^,^28^. In general, SNRs have two transcriptional activation domains in the amino (AF-1) and carboxyl (AF-2) termini. The amino-terminal domain, DNA-binding domain, hinge domain, LBD and carboxy-terminal domain all contain highly flexible regions that are important for function^29^. Their structural behaviour depends largely on the presence or absence of ligands together with their partner proteins^30^. SNRs interact with several partner proteins such as HSP90 (ref. 31) and TIF2 (ref. 32). TIF2, a transcriptional co-activator, act by bridging molecules between the receptor and the general transcription machinery and modifying chromatin within the promoter and enhancer regions by histone acetylation, methylation and phosphorylation. Following the strategy described above, we were able to stabilize the ligand-binding domain of the human estradiol (E2) nuclear receptor (ERα-E) by E2 (ref. 14), cysteine to serine mutations^13^ and/or keeping the thioredoxin fusion after cleavage^33^ (Table 1). Here we show the production and characterization of the ERβ ligand-binding domain (ERβ-EF) and the ligand-binding domain of the glucocorticoid nuclear receptor (GR-E) in complex with a 150 amino-acid long domain of the TIF2 co-activator (623–772) containing the three SNR-binding motifs.

Analysis of the sequence, structure and model of ERβ, GR and TIF2 led us to examine several constructs (Supplementary Fig. 1) that were assembled with different purification and solubility tags. The sequence alignments, secondary structure predictions, disorder analysis with IUpred, secondary structures from the Protein Data Bank and the limits of the fragments tested are represented in Supplementary Data 1–3, files generated with the programme JALVIEW^34^. Domains predictions, PONDR disorder and solubility analysis are shown in Supplementary Figs 2–4 for ERβ, GR and TIF2, respectively. The expression of ERβ was tested in *Escherichia coli* (Supplementary Fig. 5). The thioredoxin fusion for the ERβ-EF (255–530) which provided satisfactory levels of solubility was selected for further studies (the thioredoxin fusion gave the best results for ERα as described in previous results^14^). GR expression was tested in *E. coli* and insect cells. The more promising system was the HIS-NUS-GR-E (524–777) expressed in *E. coli* (Supplementary Fig. 6). To further improve the soluble expression of GR-E, a mutant was designed (GRtm-E) (Supplementary Figs 7–9). The structure of GR (PDB id: 1P93 (ref. 35)) was used as a reference structure. Two hydrophobic surface residues, tryptophan 557 and tryptophan 712 were mutated to threonine and serine, respectively. The amino acid threonine was chosen to stabilize the hydrophobic core by predicted interactions with isoleucine 747 (Supplementary Fig. 7E). A cysteine surface residue present in the native GR sequence and mutated into aspartic acid in the structure used as reference (1P93) was mutated to alanine (Supplementary Fig. 9). With this construct, satisfactory expression of soluble GRtm-E was achieved (Supplementary Fig. 10).

**Figure 3:**
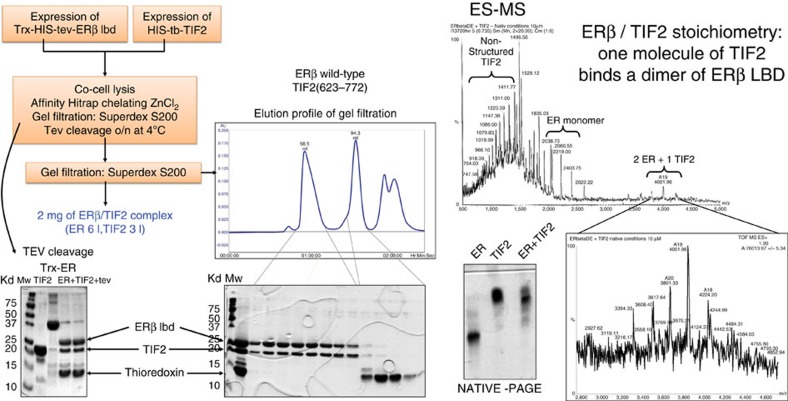
ERβ EF/TIF2 (623–772) complex purification and characterization. The two partners were produced separately. The complex was formed by co-cell lysis followed by tag cleavage and purified by size exclusion chromatography. The thioredoxin tag was well separated from the complex as shown in the SDS–PAGE on the gel filtration elution fractions. The complex was analysed and characterized by native gel electrophoresis and ES-MS. The ES-MS spectrum revealed the presence of unfolded TIF2 (623–772) (poly-charged species), monomers of ERβ LBD and dimers of ERβ LBD bound to a folded monomer of TIF2 (623–772) suggesting an induced folding mechanism upon TIF2 binding.

**Figure 4:**
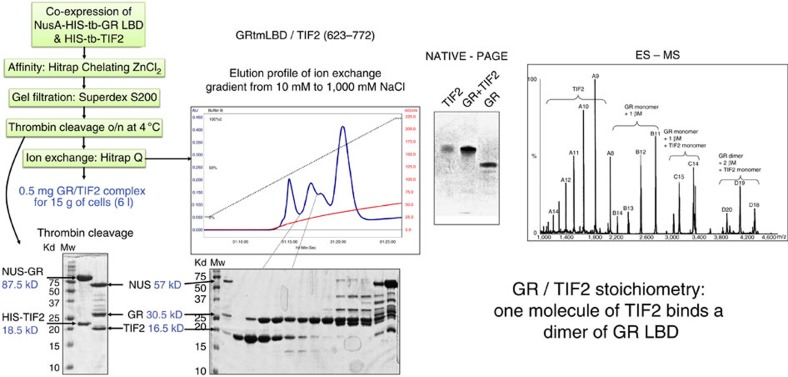
Production and characterization of the GR/TIF2 (623–772) complex. The complex was produced by co-expression of the two proteins in the same cell. After affinity chromatography and gel filtration the tags were removed by thrombin cleavage followed by ion exchange chromatography. The fractions containing the complex were used for native PAGE and ES-MS. The ES-MS spectrum shows the presence of unfolded TIF2 (623–772) (poly-charged species), monomers of GR, monomer and dimer of GR bound to one molecule of TIF2. The transition from a poly-charged species of TIF2 to structured TIF2 when bound to GR indicates that the GR induces the folding of the co-activator molecule upon GR binding.

Constructs of TIF2 containing the three NR binding motifs were designed and tested for soluble expression (Supplementary Fig. 11) in *E. coli*. The construct (623–772) with a small hexahistidine tag was selected to reconstitute the SNR/TIF2 complex. The ERβ-EF/TIF2 complex has been produced by co-cell lysis (Fig. 3). Native mass spectrometry analysis showed the presence of non-structured TIF2, monomers of ERβ-EF and dimers of ERβ-EF bound to a structured monomer of TIF2. The observation of unfolded TIF2 when alone but folded TIF2 when in complex suggests that folding is induced upon binding. For GRtm-E, co-expression in the same cell (*E. coli*) was needed to produce a stable complex. This complex was purified directly after cell lysis by affinity chromatography (Fig. 4). Native mass spectrometry analysis revealed the presence of four species. A poly-charged TIF2 characteristic of an unfolded species, free GRtm-E, TIF2 in complex with one molecule of GRtm-E and TIF2 in complex with a dimer of GRtm-E. As for ERβ this indicates that folding is induced upon TIF2 binding with a stoichiometry of 1 TIF2 for 2 GR.

### HIV-1 PICs

**Figure 5:**
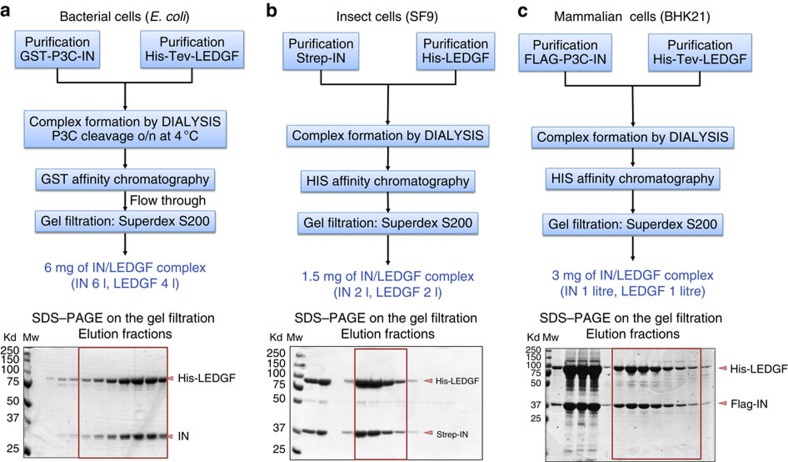
Production and purification of the HIV-1 IN and human LEDGF complex. The complex was produced in (**a**) *E. coli*, (**b**) insect and (**c**) mammalian cells. Proteins were produced and purified separately. The complex was formed by mixing the two proteins and by the removal of the solubilizing agents followed by a gel filtration chromatography. The elute fractions were analysed by SDS–PAGE.

HIV-1 PICs are dynamic and heterogeneous complexes whose composition and structure changes with time and cellular localization^36^,^37^. The core protein of the PIC, the integrase (IN) enzyme consists of three structural and functional domains, namely the N-terminal zinc binding domain (residues 1–50), the central catalytic core domain (residues 50–212) containing the D, D, E triad that coordinates divalent ions and the C-terminal domain (residues 213–288; Supplementary Fig. 12). IN interacts with several cellular and viral partners such as INI1 (ref. 38), a component of the SWI/SNF chromatin remodelling complex, or LEDGF^39^,^40^, a transcriptional co-activator. LEDGF is a 530 amino-acid long protein containing the integrase binding domain in its C-terminal moiety (Supplementary Fig. 13). It promotes efficient infection and tethers IN to favoured target sites in infected cells^41^. At the structural level, the interaction with LEDGF was shown to produce an IN active form by maintaining a stable HIV-1 IN tetramer^12^. Several partial X-ray and NMR structures of IN have been solved^42^ as well as cryoEM structures of PIC complexes^11^,^12^. Their comparison has revealed that IN displays considerable structural flexibility which accounts for its ability to interact with multiple partners and to intervene in numerous biological functions by exposing and reshaping interaction surfaces^43^,^44^,^45^. Two IN complexes participating in the PIC were produced and purified using the strategy described above (Table 1). The IN and LEDGF proteins were produced and purified separately and the complex was formed *in vitro* upon dialysis. Its CryoEM structure was solved in the presence and absence of DNA^12^. The ternary complex IN/LEDGF/INI1(174–289) was purified using the same strategy and its CryoEM structure solved in the presence of DNA^11^. Here we compare the production of HIV-1 IN and human LEDGF in *E. coli*, insect (SF9) and mammalian (BHK21) cells (Fig. 5).

**Figure 6:**
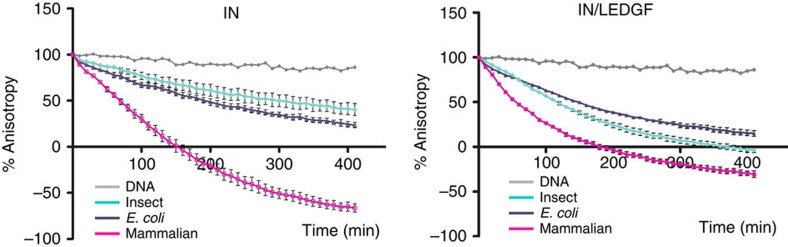
Functional 3′-processing assay. The IN and the IN/LEDGF produced in mammalian cells have a higher enzymatic activity compared to those purified from insect or *E. coli* cells.

The sequence alignments, secondary structure predictions, disorder analysis with IUpred and secondary structures from PDBs are represented in Supplementary Data 4 and 5, files generated with the programme JALVIEW^34^. Domains predictions, PONDR disorder and solubility analysis are shown in Supplementary Figs 14 and 15 for IN and LEDGF, respectively. For IN, the alignment from the Los Alamos sequence database was used (http://www.hiv.lanl.gov/). Following sequence analysis, full-length constructs were generated for IN and LEDGF. The best expression conditions (induction at 18 °C in presence of sucrose) and solubilizing buffer (1 M NaCl, 7 mM 3-[(3-cholamidopropyl)dimethylammonio]-1-propanesulfonate (CHAPS)) were assessed for expression in *E. coli* (Supplementary Figs 16–18). After expression in the three organisms, purification and complex reconstitution upon *in vitro* dialysis (Fig. 5), the solubilizing buffers (Table 2) and the 3′ processing activity of IN and the IN/LEDGF complex were tested (Fig. 6). The proteins produced in mammalian cells displayed increased solubility as compared with those produced in *E. coli* or insect cells. For the HIV-1 IN alone, the presence of detergent (CHAPS) was no longer required and the salt concentration could be reduced to 0.5 M NaCl. The 3′-processing activity was significantly enhanced when the IN or IN/LEDGF proteins were produced in the mammalian cells compared to the proteins produced in *E. coli* or insect cells. These results highlight the importance of the folding pathways and/or post-translational modifications in the structure and function of HIV-1 IN. The high quality, in terms of stability and function, of the PIC complexes produced in mammalian cell opens new perspectives for future structure–function studies.

## Discussion

We report here a standardized procedure to produce and purify stable and soluble complexes starting from unstable protein in amounts allowing *in vitro* structural and functional studies. This technology enabled us to study challenging proteins such as the human oestrogen and glucocorticoid nuclear receptors as well as the HIV-1 integrase in the context of the pre-integration complex. It allowed examination of the relationships between structure and function for ERα^13^,^14^,^33^ and HIV-1 pre-integration complexes^11^,^12^.

In this publication we present the production of large amounts of stable complexes of ERβ and GR with a 150 amino-acid long TIF2 domain containing the three NR-binding motifs (623–772) by *in vitro* complex reconstitution and co-expression respectively. Native mass spectrometry analysis showed the presence of the species ERβ_1_, ERβ_2_/TIF2_1_ as well as GR_1_, GR_1_/TIF2_1_ and GR_2_/TIF2_1_ suggesting that, in the absence of TIF2 (623–772) containing the three LXXLL motifs, ERβ and GR are in a monomeric form, whereas in the presence of TIF2 they form a LBD dimer in complex with a monomer of TIF2 and thus solve the issue of the oligomerization state of SNR LBDs^46^. Moreover we demonstrate that TIF2 (623–772) is disordered when not bound to SNRs and undergoes induced folding upon binding to its partner SNR. For a full understanding of SNR action, structures of disordered regions like the A/B domain or full-length SNRs in complex with disordered regions of co-activator and/or corepressor proteins will be necessary. In this work we set up a methodology which will allow us to reach this goal.

The IN/LEDGF complex was produced by *in vitro* complex reconstitution in prokaryotic and eukaryotic cells. For HIV-1 IN we show that production in mammalian cells leads to increased solubility and 3′-processing activity compared with IN produced in *E. coli* or insect cells. This demonstrates the influence of the organism used for expression and implies that distinct folding pathways and/or post-translational modifications are required for the structure and function of HIV-1 integrase and its complex with LEDGF. The new procedure we describe here will provide a powerful research tool for structural and functional studies of flexible proteins participating in non-covalent macromolecular complexes.

## Methods

### Bioinformatics analysis

Sequence alignments were performed using PipeAlign^15^ software. followed by disorder analysis (PONDR-FIT^16^), domain prediction (CDD^18^), solubility predictions (SOLpro in SCRATCH^19^ programme suite and PROSO^20^) and secondary structure predictions (JPRED^21^). The GR LBD structure (PDB id: 1P93) was used to analyse the GR surface electrostatic potential for hydrophobic patches. The electrostatic potential was calculated using the coulombic surface colouring option in Chimera software^23^. The mutant structures were generated in Coot^24^ software. The result is shown in supplementary Figs 7–9. Three surface residues were mutated: Trp557 to Thr, Cys 636 to Ala and Trp712 to Ser.

### Preparation of expression plasmids and viruses

*Expression in E. coli*. The cDNAs encoding the proteins of interest were cloned in pENTR vectors (Invitrogen) and transferred in pET expression plasmids^47^ using the Invitrogen Gateway strategy. The expression vectors were then transferred into *E*. *coli* BL21(DE3) host strain (Invitrogen) for protein expression.

*Expression in insect cells*. The cDNAs encoding the proteins of interest were transferred into strep-tag or his-tag expression vectors^48^ respectively using the Invitrogen Gateway strategy. Expression vectors were then transferred into *E. coli* DH10Bac (Invitrogen) to generate the recombinant bacmids. Bacmids were purified using a standard alkaline lysis and isopropanol precipitation method, and subsequently transfected to SF9 insect cells. After 10 days culture, cell lysis occurred and viral particles were recovered from the culture supernatant. To obtain a high titre virus, a round of viral amplification was performed by infecting SF9 cells at a multiplicity of infection of 0.1, and cultivating them until lysis. Supernatants were kept at 4 °C.

*Expression in mammalian cells*. HisLEDGF and FlagIN were expressed in Baby Hamster Kidney suspension cells (BHK21-C13-2P, Sigma-Aldrich) using a vaccinia virus gateway expression system^49^,^50^. Briefly, LEDGF and IN coding sequence were fused in frame with N-terminal 6His and Flag tags, respectively. Expression plasmids were then integrated to the Modified Vaccinia Ankara (MVA) vector encoding a T7 RNA polymerase. Recombinant viruses: MVA-T7-HisLEDGF and MVA-T7-FlagIN were used for protein production upon infection of BHK 21 cells.

### Small-scale expression tests

Small-scale expression tests were performed in volumes from 5 to 50 ml of *E. coli*, insect or mammalian cell culture. Cells were harvested and lysed by sonication in the appropriate buffer. After centrifugation, the soluble and total extracts were deposited on a SDS–PAGE denaturing gel. The intensity of the protein band in the soluble fraction compared to the total fraction was used to define the propensity of a given construct to increase the solubility and stability of the expressed protein or complex.

The level of expression and solubility was estimated on SDS–PAGE gels stained with Coomassie blue. The intensity of the band corresponding to the protein of interest was compared with the total amount of protein. In the results shown in Supplementary Figs 3, 6, 9 and 10 the gradation corresponds to the absence of expressed protein (−) up to 50% of the total amount of protein (++++). The total extract represents the total amount of protein expressed and the soluble extract obtained after removal of the non-soluble fraction after centrifugation represents the amount of soluble protein.

### Human ERβ-EF / TIF2 (255–530) production

Trx-His-tev-ERβ-EF (255–530) was expressed in *E. coli* BL21(DE3) cells cultured in lysogeny broth (LB) medium supplemented with 10% (w/v) sucrose, 100 mg l^−1^ ampicillin and 10 μM estradiol (E2) at 37 °C until the OD reached 0.5. The culture was then slowly cooled to 18 °C before adding 0.5 mM IPTG (isopropyl-β-D-thiogalactoside) and left overnight. His-tb-TIF2 (623–772) was expressed in *E. coli* BL21(DE3) cells grown in LB medium supplemented with 10% (w/v) sucrose and 100 mg l^−1^ kanamycin at 37 °C until the OD reached 0.5. The culture was then slowly cooled to 20 °C before adding 0.5 mM IPTG and left overnight. All the following purification steps were performed at 4 °C. The protein purity was followed on SDS–PAGE and protein concentrations were measured by UV absorption at 280 nm. The cells were harvested by centrifugation and suspended in 50 mM Phosphate Na/K pH 7.5, 50 mM NaCl, 10 mM β-mercaptoethanol, 10 μM E2. Cells from 1 l of ERβ-EF and 1 l of TIF2 were mixed and lysed by sonication. The extract was centrifuged at 100,000*g* for 60 min. The crude extract was loaded on a 5 ml ZnCl_2_ affinity column. After washing out nonspecifically bound material, proteins were eluted with 15 column volumes of a linear gradient (0–100 mM imidazole). The fractions corresponding to the elution peak were mixed and concentrated on a Centriprep with a cutoff of 10 kD followed by a gel filtration chromatography (Superdex 200) in a buffer containing 50 mM Tris pH7.5, 250 mM NaCl, 10 mM β-mercaptoethanol and 10 μM E2. To remove the tag, TEV protease was added and the sample was kept overnight at 4 °C. The sample was then loaded on a Superdex G200 gel filtration column and the elution peak was concentrated on a Centriprep (Milllipore) with a cutoff of 10 kD. Starting from 1 l of each culture we obtained 10 mg of ERβ-EF/TIF2 (255–530) complex at 12 mg ml^−1^.

### Human GRtm LBD/TIF2 (255–530) production

The NusA-(His)6-thrombin-GR_LBD triple mutant C638A, W557T and W712S (residue 524–777) was co-expressed with (His)6-thrombin-TIF2 (623–772) in *E. coli* BL21(DE3) cells. The culture was grown at 18 °C in LB medium supplemented with 10%(w/v) sucrose, 100 mg l^−1^ ampicillin, 50 mg l^−1^ kanamycin in presence of dexamethasone. All purification steps were performed at 277 K. The protein purity was analysed by SDS–PAGE and protein concentrations were measured by UV absorption at 280 nm. The cells were sonicated on ice in 50 mM Na/K phosphate buffer pH7.5, 250 mM NaCl, 10 μM dexamethasone, 10 mM β-mercaptoethanol buffer and the extract was centrifuged at 100,000*g* for 1 h. The purification was performed in a three-step procedure in the presence of dexamethasone: zinc affinity, gel filtration and anion exchange chromatography. The crude extract was loaded onto a 5 ml zinc affinity column (Hitrap Chelating, GE Healthcare). Nonspecifically bound proteins were removed by a 10 column volumes wash in a 50 mM Na/K pH7.5 phosphate, 250 mM NaCl, 10 μM dexamethasone and 10 mM β-mercaptoethanol buffer. Elution was performed in a 15 column volumes imidazole gradient from 0 up to 0.5 M. The sample was then concentrated on Centriprep (Millipore) with a cutoff of 30 kD and analysed on SDS–PAGE. The complex was further purified on a gel filtration Superdex 200 column (GE Healthcare) equilibrated in a 50 mM Tris pH8.0, 250 mM NaCl, 10 μM dexamethasone and 10 mM β-mercaptoethanol buffer. Endoproteolytic cleavage of the fusion proteins was achieved using one unit of thrombin (Sigma) per milligram of fusion substrate and incubating at 4 °C overnight. The completeness of the proteolytic reaction was assessed by SDS–PAGE. Following the digestion step, the sample was diluted and further purified by anion exchange chromatography on Hitrap Q (GE Healthcare). The column was equilibrated in 10 mM TRIS pH8.5, 10 mM NaCl, 10 μM dexamethasone and 5 mM β-mercaptoethanol buffer. A salt gradient from 10 mM to 1 M NaCl over 20 column volumes was used for elution. Peak fractions corresponding to the complex were pooled and concentrated by ultrafiltration. With this procedure we obtained 0.5 mg complex from a 3 l culture.

### Mass spectrometry analysis

All studies were performed using an electrospray time-of-flight mass spectrometry (ESI-TOF) mass spectrometer (LCT, Micromass, Manchester). The protein samples were submitted to buffer exchange in 50 mM ammonium acetate pH 8.5, 10 μM estratiol for ERβ or 10 μM dexamethasone for GR. The sample was continuously infused into the ion source at a flow rate of 4 μl min^−1^ using a Harvard Model 11 syringe pump (Harvard Apparatus).

### HIV-1 integrase/human LEDGF production

*Production in E. coli*. The IN/LEDGF complex was produced as previously described^12^. Briefly, GST-P3C-integrase and His-Tev-LEDGF were expressed in *E. coli* BL21 DE3 pRARE cells overnight at 18 °C in LB medium supplemented with 10% sucrose. Proteins were solubilized and purified in 50 mM Hepes pH7.5; 1 M NaCl; 7 mM CHAPS; 5 mM MgCl_2_; 2 mM β-mercaptoethanol buffer. Purified partners were then mixed and the complex was formed upon removal of solubilizing agents through dialysis. Addition of P3C protease at the beginning of the dialysis allowed removal of the GST tag from IN. Free GST tag was removed by GST affinity purification, the complex was recovered from the flow-through and submitted to subsequent nickel affinity chromatography. A final purification step was performed on a Highload 16/60 superdex 200 prep grade gel filtration column (GE Healthcare).

*Production in insect cells*. SF21 insect cells, maintained in Erlen-meyer flasks with agitation (250 r.p.m.) were grown in suspension in Sf-900 II synthetic medium to a density of 0.8 × 10^6^ cells per ml and infected at a multiplicity of infection of 1. Forty-eight hours after infection, cells were harvested by centrifugation (20 min at 400*g*), washed with cold PG buffer (PSB 1 × , 10% glycerol), pelleted and flash frozen in liquid nitrogen. Cell pellets were stored at −20 °C.

Cells were harvested in 30 ml lysis buffer (50 mM Hepes, pH 7.5; 1 M NaCl; 2 mM MgCl_2_; 2 mM β-mercaptoethanol with 1.5 mM CHAPS for strep-tag purification or 7 mM CHAPS for his-tag purification) per 10^9^ cells and lysed through sonication (Bioblock Scientific VibraCell sonicator with 13 mM probe, 40% intensity, 2 s pulses, 1 min sonication per 10^9^ cells). Lysates were clarified by centrifugation (1 h, 100,000*g*, 4 °C) and loaded on the appropriate affinity column (GE Healthcare StrepTrap HP 5 ml or HisTrap FF crude 5 ml) after equilibration in lysis buffer. Unbound proteins were washed away with lysis buffer and protein of interest was eluted. The elution of strep-tagged Integrase was performed in a single step using lysis buffer supplemented with 2.5 mM desthiobiotin (IBA). His-tagged LEDGF was eluted by a gradient of imidazole (SIGMA) (0 to 500 mM imidazole in lysis buffer). Eluted fractions were analysed by SDS–PAGE and fractions of interest were pooled, concentrated on Amicon Ultra 15 30 kDa (Millipore) and further purified by gel filtration on a Highload 16/60 superdex 200 prep grade column (GE Healthcare) equilibrated in a 50 mM Hepes, pH 7.5; 1 M NaCl; 2 mM MgCl_2_; 2 mM βmercaptoethanol with 7 mM CHAPS buffer. Fractions of interest were stored at −20 °C after addition of 20% glycerol.

Purified partners were mixed in an IN/LEDGF ratio of 2/1.2 and complex was reconstituted by slowly removing the solubilizing agents through dialysis (Spectra/Por dialysis membrane from SpectrumLabs) against a 50 mM Hepes pH7.5; 0.25 M NaCl; 5 mM MgCl2; 2 mM β-mercaptoethanol buffer (Buffer B). Complex was then purified by nickel affinity chromatography (HisTrap FFcrude 5 ml column from GE Healthcare) and gel filtration (Highload 16/60 superdex 200 prep grade column from GE Healthcare).

*Production in mammalian cells*. Protein expression was performed using BHK21-C13-2P cells infected with 2 PFU per cell of either MVA-T7-HisLEDGF or MVA-T7-FlagIN, and was induced with 0.1 mM IPTG. The cultures were maintained on an orbital shaker at 37 °C in a humidified atmosphere containing 5% CO_2_. Twenty-four hours later, infected/induced-cells were harvested. His-tagged LEDGF expressing cell culture pellet was resuspended in 50 ml lysis buffer (1 M NaCl, 7 mM CHAPS, 25 mM Hepes (pH7.5), 5 mM MgCl_2_ and 2 mM βmercaptoethanol) containing 5 mM Imidazole, lysed by sonication and clarified by centrifugation at 100,000*g* for 45 min at 4 °C. The supernatant was loaded on a 5 ml HiTrap-Ni column (GE Healthcare). After washes, the protein was eluted by a linear gradient of imidazole (from 15 to 500 mM) and the LEDGF containing fractions were pooled. In parallel, a Flag-tagged IN expressing pellet was lysed in 25 ml lysis buffer, sonicated and clarified. The supernatant was incubated with ANTI-FLAG M2 Affinity Gel (Sigma-Aldrich) for 4 h at 4 °C with gentle shaking, after washes the protein was eluted with 16.5 mg FLAG peptide (DYKDDDDK).The eluted IN was then subjected to gel filtration (HiLoad 16/60 Superdex 200 prep grade column, GE Healthcare). The IN containing fractions were pooled. Finally the mammalian HisLEDGF-FlagIN complex was generated by mixing a portion of purified LEDGF pool with a portion of gel filtrated IN pool at a 1:1 molar ratio. The mixture was slowly dialyzed against buffer containing 500 mM NaCl, 25 mM Hepes (pH 7.5), 5 mM MgCl_2_ and 2 mM β-mercaptoethanol. The LEDGF-IN complex was harvested and concentrated using Amicon Ultra, 10 kD molecular weight cutoff device (Millipore).

As a control for an enzyme activity test, a recombinant MVA-T7 virus expressing Flag-TRN-SR2 was also generated and the target protein was purified on ANTI-FLAG M2 Affinity Gel (Sigma-Aldrich) in the same way.

### Solubility assays for purified protein

To assess the solubility limits of proteins, samples were loaded on an Amicon Ultra concentrator with appropriate cutoff and concentrated up to the appearance of a precipitate. The supernatant was centrifuged and protein concentration was determined by measuring the OD at 280 nm using the calculated extinction coefficient of each protein.

### Functional 3′-processing assay

*Oligonucleotide preparation*. Fluorescein-labelled double-stranded DNAs were prepared as follows. A forward oligonucleotide with a covalently attached 6-carboxyfluorescein (6-FAM) moiety on its 3′-end was mixed with the unlabelled complementary strand in annealing buffer (10 mM BisTris (pH 6.5), 50 mM NaCl) in a 1:1 molar ratio, the mixture was heated to 90 °C for 10 min, and annealing was allowed by slowly cooling to 4 °C. Annealing was controlled on native gels. Two 3′-6-FAM-labelled dsDNA were generated: a 40-mer oligo mimicking the U5 end of HIV-1 DNA, that is, viral DNA or vDNA, and a 49-mer random DNA sequence as a control. Unlabelled and labelled single-stranded DNA were respectively purchased from Sigma and IBA GmbH . Viral DNA (U5): 5′- GACTACGGTTCAAGTCAGCGTGTGGAAAATCTCTAGCAGT [6-FAM]-3′; 5′- ACTGCTAGAGATTTTCCACACGCTGACTTGAACCGTAGTC -3′. Random DNA: 5′- AGTTAAGTGCTGAATTATGATAGTAATCAAT ATCTACTCCTAACCTCTT [6-FAM]-3′; 5′- AAGAGGTTAGGAGTAGATATTGATTACTATCATAATTCAGCACTTAACT -3′.

*Enzymatic assays*. Fluorescence anisotropy assays were used to evaluate the 3′-processing activity of HIV-1 integrase as described^11^. Briefly, the reaction was done in a 96-well plate. One well contained 100 μl of reaction mix composed of 10 mM NaCl, 25 mM BisTris pH 6.5, 10 mM MgCl2, 5 mM DTT, 50 nM DNA and 200 nM of protein complex. The DNA is a 40 base pair double-stranded DNA, mimicking the U5′ end of HIV-1 DNA and 3′-modified by 6-fluorescein. After homogenization, 50 μl of paraffin oil were added on the top of the well to avoid evaporation. Fluorescence anisotropy measurements were performed on a PHERAstarPlus (BMGLab) spectrophotofluorimeter with an excitation polarized wavelength of 470 nm. The reaction was monitored for 6 h at 37 °C.

## Additional information

**How to cite this article:** Levy, N. *et al*. Production of unstable proteins through the formation of stable core complexes. *Nat. Commun.* 7:10932 doi: 10.1038/ncomms10932 (2016).

## Supplementary Material

Supplementary Information Supplementary Figures 1-18

Supplementary Data Set 1 Human ERβ Sequence multi alignment and domains predictions

Supplementary Data Set 2 Human GR Sequence multi alignment and domains predictions

Supplementary Data Set 3 Human TIF2 Sequence multi alignment and domains predictions

Supplementary Data Set 4 HIV1 Integrase Sequence multi alignment and domains predictions

Supplementary Data Set 5 Human LEDGF Sequence multi alignment and domains predictions

## Figures and Tables

**Figure 1:**
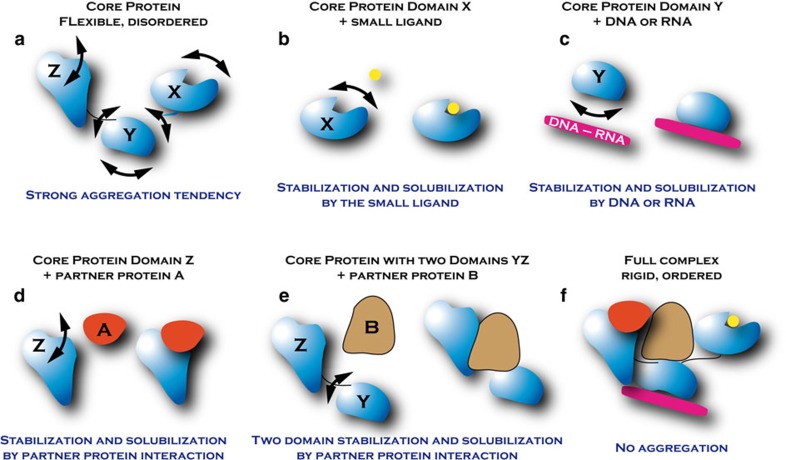
Stabilization of flexible proteins. (**a**) Multi-domain proteins participating in large macromolecular complexes, with inter and/or intra domains flexibility, have a strong aggregation tendency. To characterize structurally and functionally the entire biological complex (**f**), the protein can be divided into domains which can be stabilized by small ligands (**b**), DNA/RNA (**c**) and partner proteins (**d**,**e**).

**Table 1:** Proteins complexes produced using the strategy described.

	**Protein**	**Stabilized by**	**Strategy**	**References**
Human steroid nuclear receptors	ERα-E	Ligand (oestrogen)	Ligand in cell culture	14
		Thioredoxin fusion	Keep Thioredoxin (TRX) fusion for crystallization	33
		3Cys to Ser mutations	Mutations mimic the conformational changes induced by the ligand	13
	ERβ-EF	TIF2 (623–772)	*In vitro* reconstitution (co-cell lysis)	This article
	GRtm-E	TIF2 (623–772) and solubility mutant	In cell co-expression	This article
				
HIV-1 pre-integration complexes	IN	hLEDGF	*In vitro* reconstitution (dialysis)	12
		hLEDGF/hINI1(174–289)		11
		hLEDGF	Expression in *E. coli*, insect and mammalian cells and *in vitro* reconstitution	This article

ER, human estradiol (E2) nuclear receptor; IN, integrase; TIF2, transcriptional intermediary factor 2.

**Table 2:** IN solubility was tested in different solvents.

**IN solubility (mg ml^−1^)**	**1 M NaCl 7 mM CHAPS**	**1 M NaCl**	**0.5 M NaCl**
*E. coli*	4.0–5.0	0.1–0.5	0.1–0.3
Insect	4.0–5.0	0.1–0.5	0.1–0.3
Mammalian	4.0–5.0	2.0–3.0	2.0–3.0

IN, integrase.

The proteins produced in mammalian cells showed increased solubility compared to the *E. coli* and insect cell production.
