# Supplementary material for: Production of unstable proteins through the formation of stable core complexes
Source: Nat Commun. 2016 Mar 17;7:10932. doi: 10.1038/ncomms10932 (PMC4800440; doi:10.1038/ncomms10932)
Supplement: Supplementary Information — Supplementary Figures 1-18 [file ncomms10932-s1.pdf]

Production of unstable proteins through the formation of stable core complexes

Nicolas Levy*, Sylvia Eiler*, Karine Pradeau-Aubreton, Benoit Maillot, François Stricher¶, Marc Ruff

IGBMC, UDS, CNRS, INSERM, 1 rue Laurent Fries 67404 Illkirch

*These authors contributed equally to this work

¶Present address: Global Bioenergies, 5 rue Henri Desbruères, 91000 Evry, France

Correspondence: M.F. (email)

Supplementary figures

Supplementary figure 1

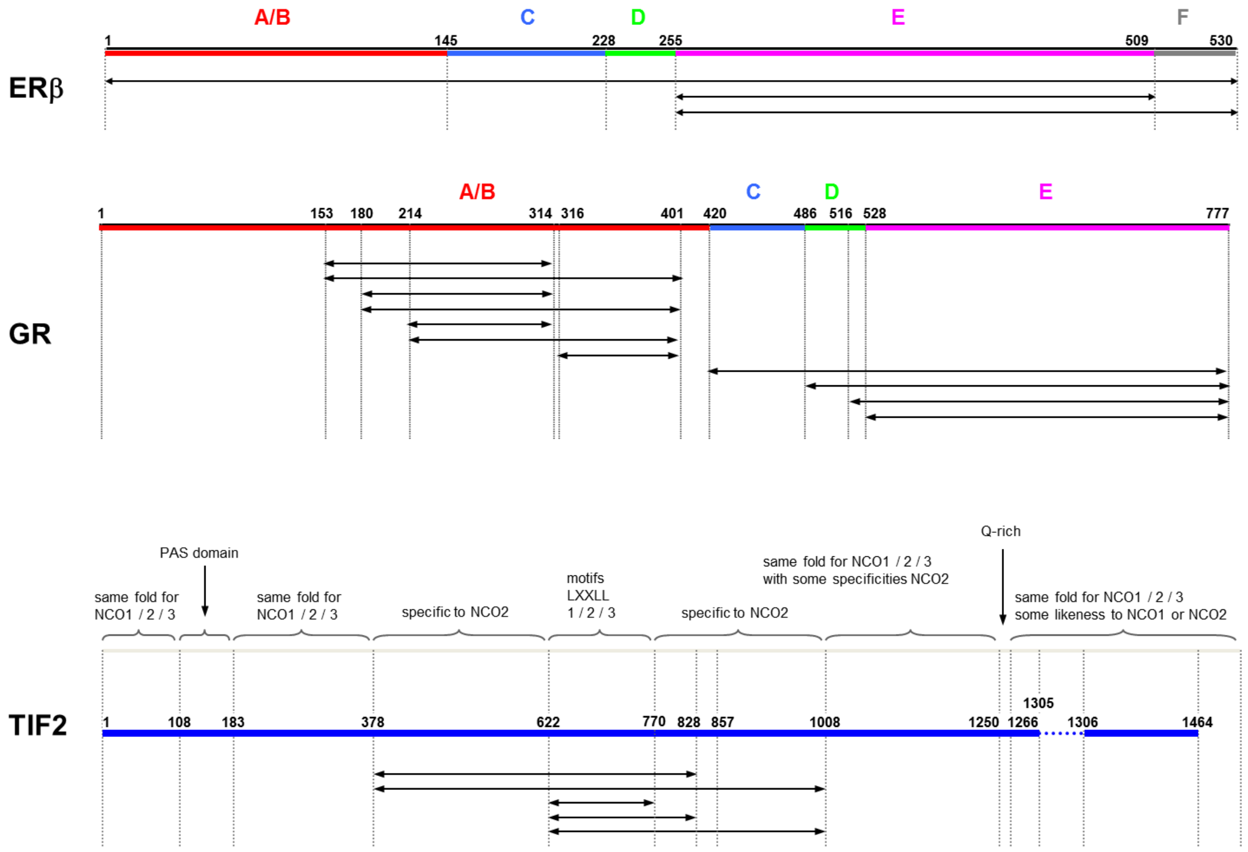

Domain organization and regions cloned for ERβ, GR and TIF2

ERβ, GR and TIF2 domains are labeled according to homologies with published sequences. Arrowed lines underneath the genes indicate the domains that were cloned and expressed.

Supplementary figure 2

CDD Domains predictions

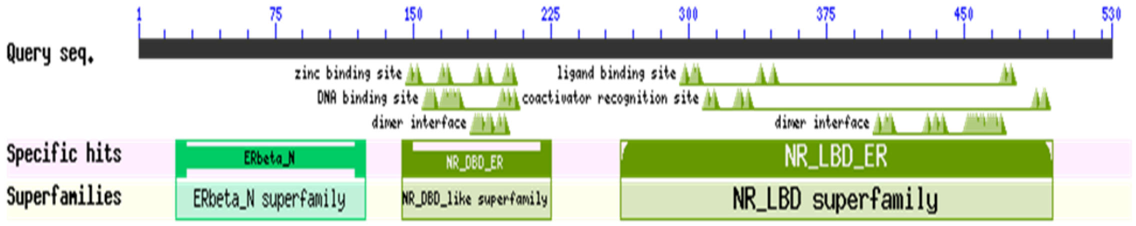

PONDR disorder analysis

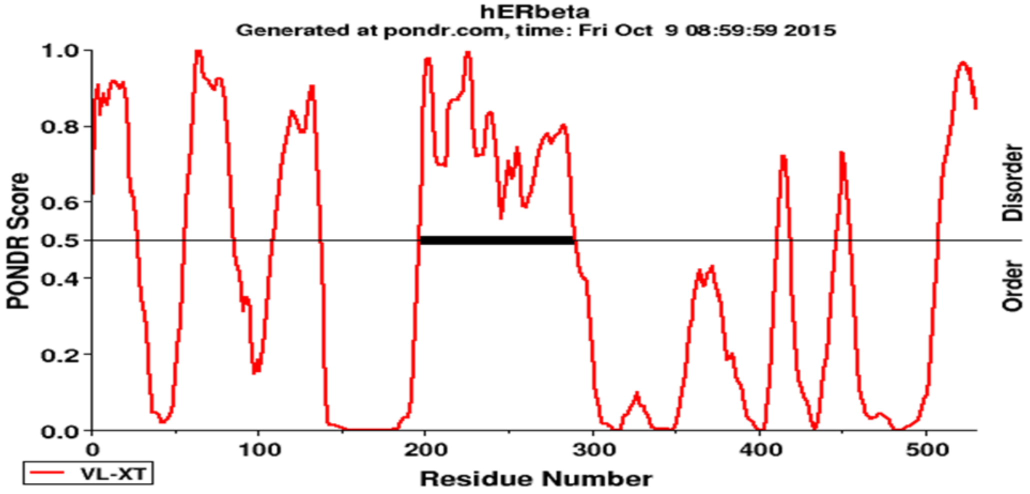

PROSO II and SOLPRO solubility predictions

Protein	PROSO II Predicted class	PROSO II Solubility score	SOLPRO Predicted solubility	SOLPRO Probability
hERbeta-Full -Length	insoluble	0.551	insoluble	0.578
hERbeta-255-530	insoluble	0.554	soluble	0.738
hERbeta-255-509	insoluble	0.435	soluble	0.660

hERβ Domains prediction with CDD, disorder analysis with PONDR and solubility predictions with PROSO II and SOLPRO

Supplementary figure 3

CDD Domains predictions

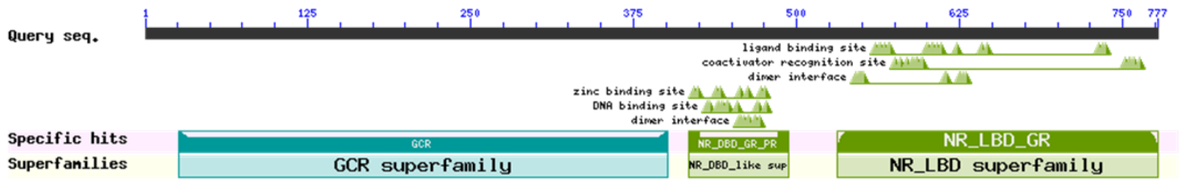

PONDR disorder analysis

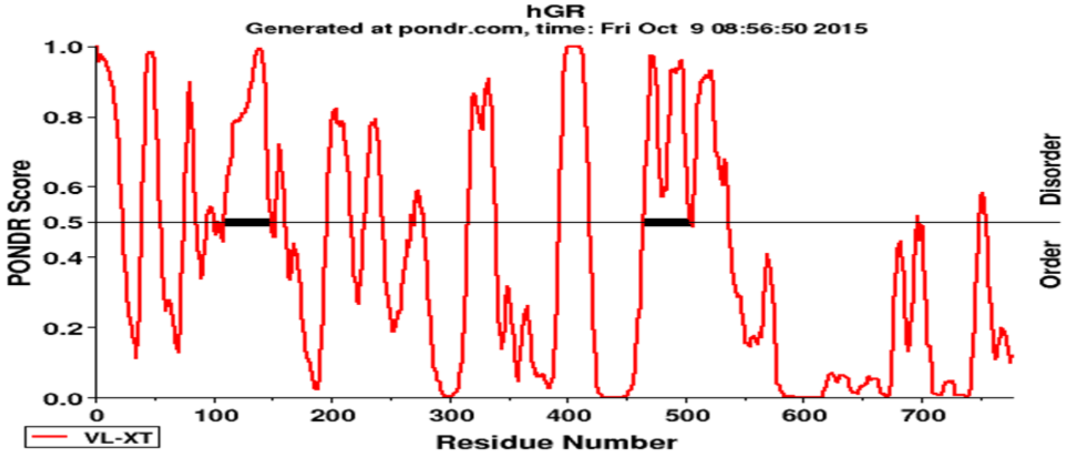

PROSO II and SOLPRO solubility predictions

Protein	PROSO II Predicted class	PROSO II Solubility score	SOLPRO Predicted solubility	SOLPRO Probability
hGR-Full –Length	insoluble	0.592	soluble	0.640
hGR-420-777	insoluble	0.509	insoluble	0.806
hGR-486-777	insoluble	0.522	insoluble	0.628
hGR-516-777	soluble	0.668	insoluble	0.694
hGR-524-777	soluble	0.631	insoluble	0.695

hGR Domains prediction with CDD, disorder analysis with PONDR and solubility predictions with PROSO II and SOLPRO

Supplementary figure 4

CDD Domains predictions

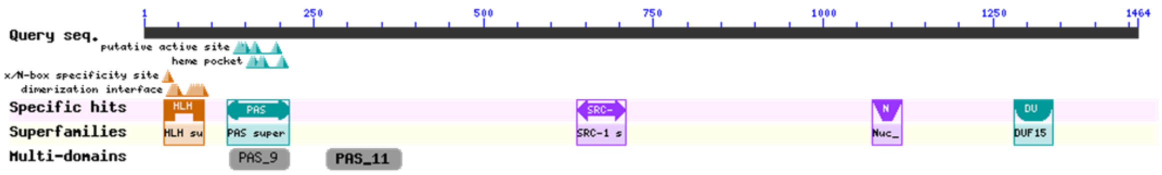

PONDR disorder analysis

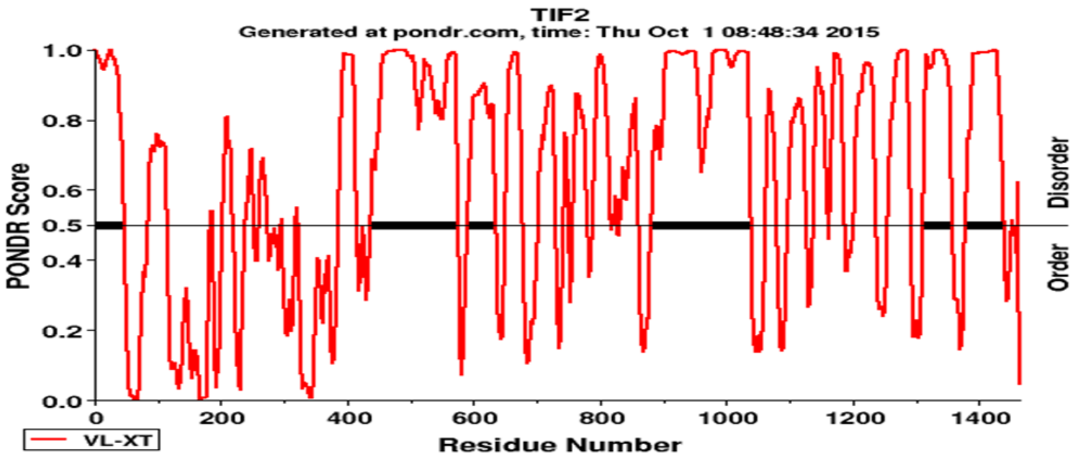

PROSO II and SOLPRO solubility predictions

Protein	PROSO II Predicted class	PROSO II Solubility score	SOLPRO Predicted solubility	SOLPRO Probability
hTIF2-Full –Length	insoluble	0.511	insoluble	0.754
hTIF2-623-772	soluble	0.824	soluble	0.933
hTIF2-623-828	soluble	0.793	soluble	0.952
hTIF2-623-1008	soluble	0.629	soluble	0.688
hTIF2-378-828	soluble	0.716	soluble	0.930
hTIF2-378-1008	soluble	0.625	soluble	0.602

hTIF2 Domains prediction with CDD, disorder analysis with PONDR and solubility predictions with PROSO II and SOLPRO

Supplementary figure 5

ERβ	HIS		GST		HIS-TRX		HIS-MBP	
	LBS/18°C		LBS/18°C		LBS/18°C		LBS/18°C	
	T	S	T	S	T	S	T	S
FL 1 – 530	**++**	**-**	**++**	**-**	**++**	**-**	**++**	**-**
EF 255 – 530	nd	nd	nd	nd	**+++**	**++**	nd	nd

Expression and solubility of a full length ERβ or the ligand binding domain tagged in fusion with His, GST, TRX and MBP. T: Total Extract, S: Soluble extract, LBS: LB medium for cell culture plus 10% sucrose, nd: not done. The full length ERβ was tested in expression in *E. coli* with HIS, GST, Thioredoxin (TRX) and MBP fusion. The ERβ LBD EF was tested in *E. coli* with the TRX fusion. The His-Trx-ERβ LBD yielded soluble expression. The optimized buffer for protein solubilization is 300 mM KCl, 100 mM Phosphate buffer pH=7.5, 10 mM βmercaptoethanol. The level of expression and solubility was estimated on SDS page stained with coomassie blue. The intensity of the band corresponding to the protein of interest was compared to the total amount of proteins. The gradation corresponds to the absence of expressed protein (-) up to 50% of the total amount of proteins (++++).

Supplementary figure 6

GR expression tests in *E. Coli*

GR (*E. coli*)	HIS				FLAG				HIS-NUS			
	LBS/37°C		LBS/18°C		LBS/37°C		LBS/18°C		LBS/37°C		LBS/18°C	
	T	S	T	S	T	S	T	S	T	S	T	S
CDE 420 – 777	**+/-**	**-**	**-**	**-**	**+**	**-**	**-**	**-**	**++**	**-**	**++**	**+**
DE 486 – 777	**++**	**-**	**-**	**-**	**++**	**-**	**+**	**-**	**++**	**-**	**++**	**++**
E 516 – 777	**+/-**	**-**	**+/-**	**-**	**++**	**-**	**+**	**+/-**	**++**	**-**	**++**	**+**
E 524 – 777	**-**	**-**	**-**	**-**	**+**	**-**	**++**	**-**	**++**	**-**	**++**	**+**

Expression tests for 4 constructs of GR in fusion with His, Flag and NUS. T: Total Extract, S: Soluble extract, LBS: LB medium for cell culture plus 10% sucrose. Only the NUS fusion expressed in LBS medium at 18°C yielded soluble protein. Solubilization buffer: 250 mM NaCl, 50 mM Tris pH7.5, 10 µM dexamethasone. The intensity of the band corresponding to the protein of interest was compared to the total amount of proteins. The gradation corresponds to the absence of expressed protein (-) up to 50% of the total amount of proteins (++++).

GR expression tests in Insect cells

GR (insect)	HIS		GST	
	T	S	T	S
CDE 420 – 777	**+**	**-**	**++**	**+/-**
DE 486 – 777	**nd**	**nd**	**++**	**+/-**
E 516 – 777	**+**	**-**	**++**	**+/-**
E 524 – 777	**+**	**-**	**nd**	**nd**

Expression tests for 4 constructs of GR in fusion with His and GST. T: Total Extract, S: Soluble extract, nd: not done. No tested constructs enabled soluble proteins expression. Solubilization buffer: 250 mM NaCl, 50 mM Tris pH7.5, 10 μM dexamethasone. The intensity of the band corresponding to the protein of interest was compared to the total amount of proteins. The gradation corresponds to the absence of expressed protein (-) up to 50% of the total amount of proteins (++++).

Supplementary figure 7

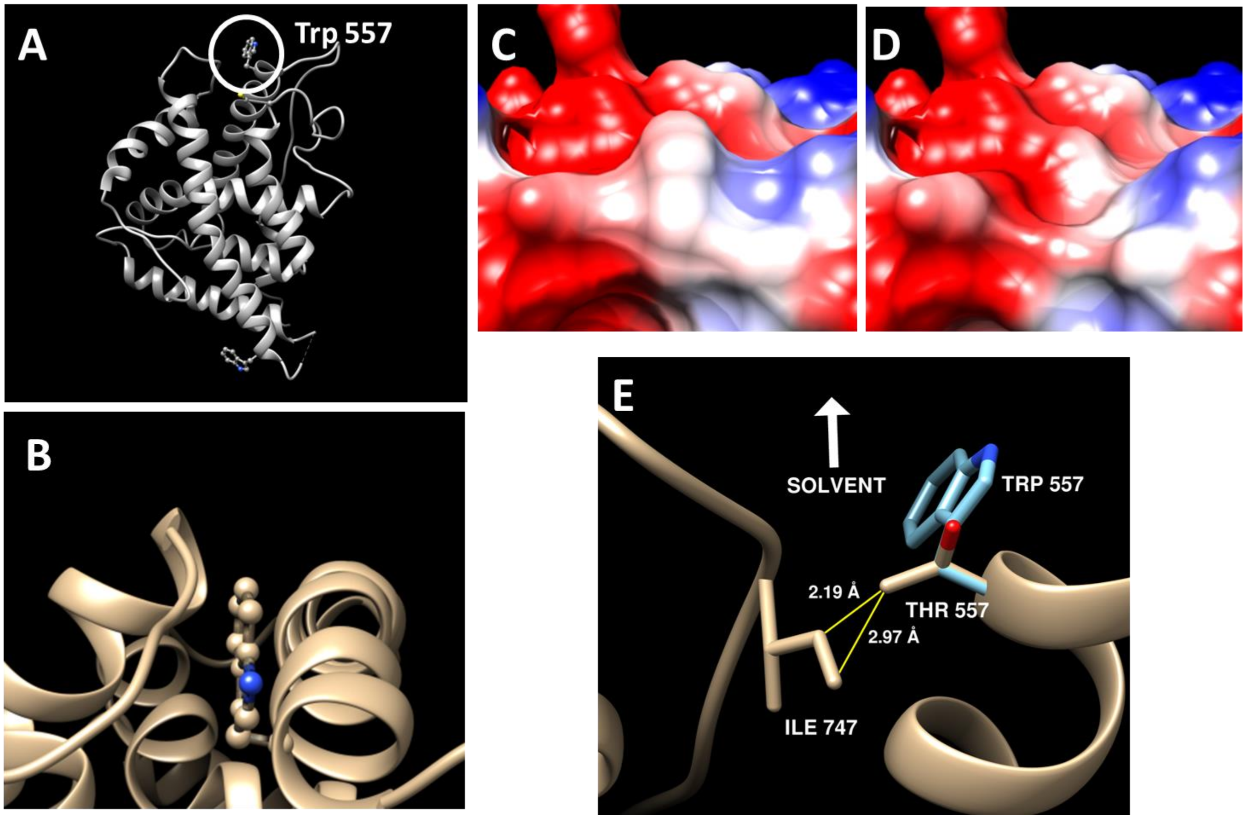

GR-LBD surface mutations to improve solubility: Trp 557 to Thr

A) Full view of the GR LBD reference structure (PDB: 1P93). The residue Trp 557 is circled in white. B) Close up view of Trp 557. C) Surface electrostatic potential representation. Positive charges are in blue, negative charge in red and hydrophobic surface in grey. The potential was calculated using the coulombic surface coloring option in Chimera. D) Surface potential after Trp → Thr mutation. E) Superposition of the wild type structure (blue) and the Thr to Trp mutant (grey).

The residue Trp 557 was chosen to be mutated primarily to reduce hydrophobicity at the surface of the protein and secondary we choose to mutate it in threonine which creates hydrophobic interaction with isoleucine 747 and direct its hydroxyl group towards the solvent combining two effects: (1) increasing surface hydrophilicity and (2) stabilizing the hydrophobic core.

Supplementary figure 8

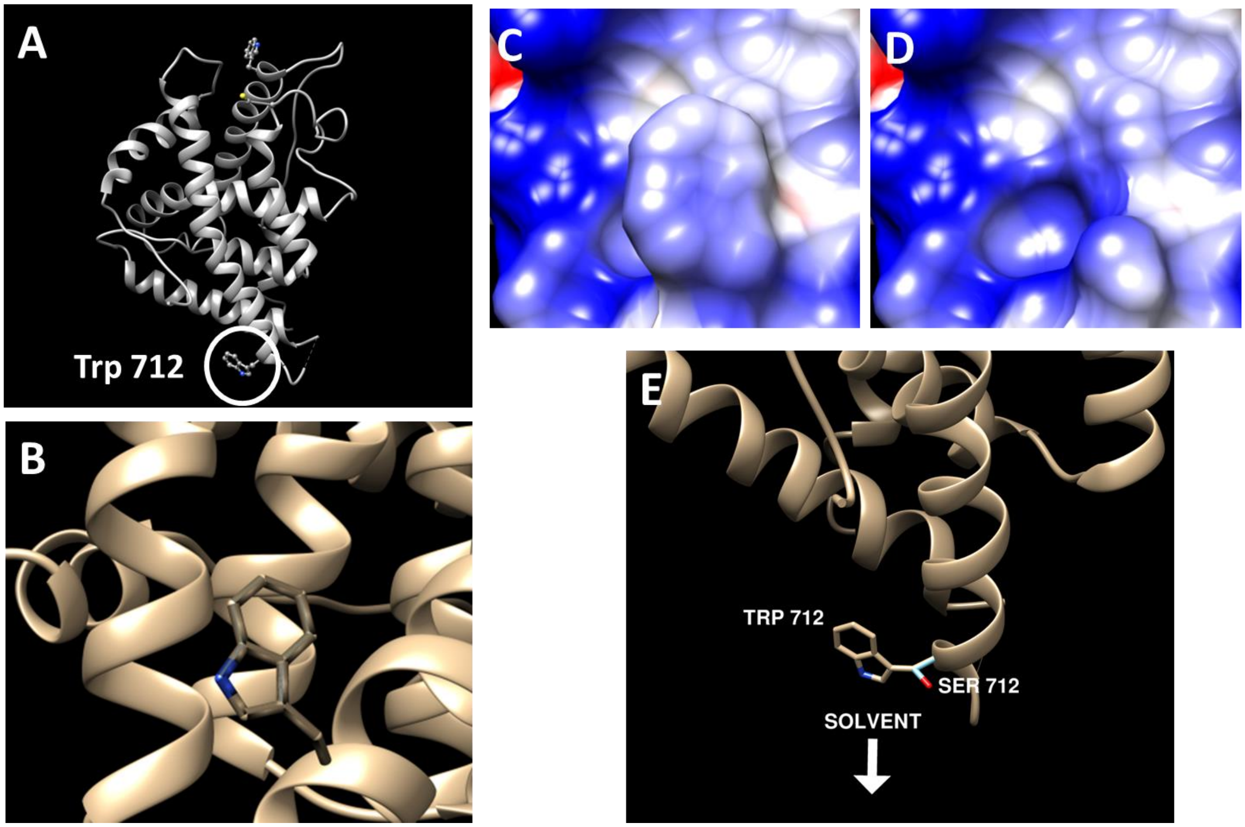

GR-LBD surface mutations to improve solubility: Trp 712 to Ser

A) Full view of the GR LBD structure (PDB: 1P93). The residue Trp 712 is circled in white. B) Close up view of Trp 712. C) Surface electrostatic potential representation. Positive charges are in blue, negative charge in red and hydrophobic surface in grey. The potential was calculated using the coulombic surface coloring option in Chimera. C) Surface potential after Trp → Ser mutation. Superposition of the wild type structure (blue) and the Thr to Ser mutant (grey).

Supplementary figure 9

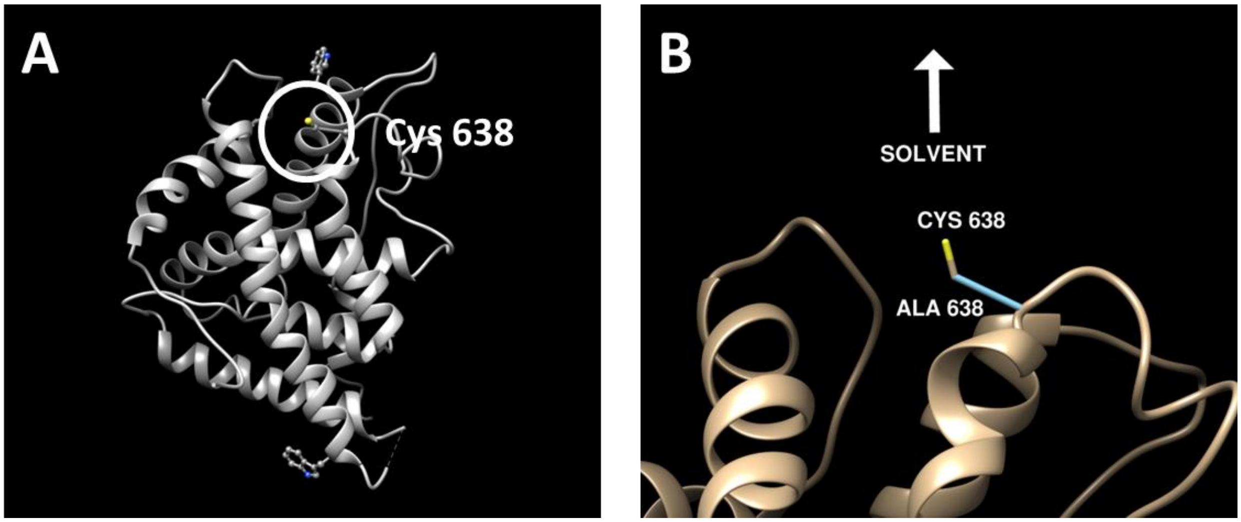

GR-LBD surface mutations to improve solubility: Cys 638 to Ala

A) Full view of the GR LBD structure (PDB: 1P93). The residue Cys 638 is circled in white. In the 1P93 structure, this residue was mutated in Asp. B) Superposition of the wild type structure (grey) and the Cys to Ala mutant (blue).

Supplementary figure 10

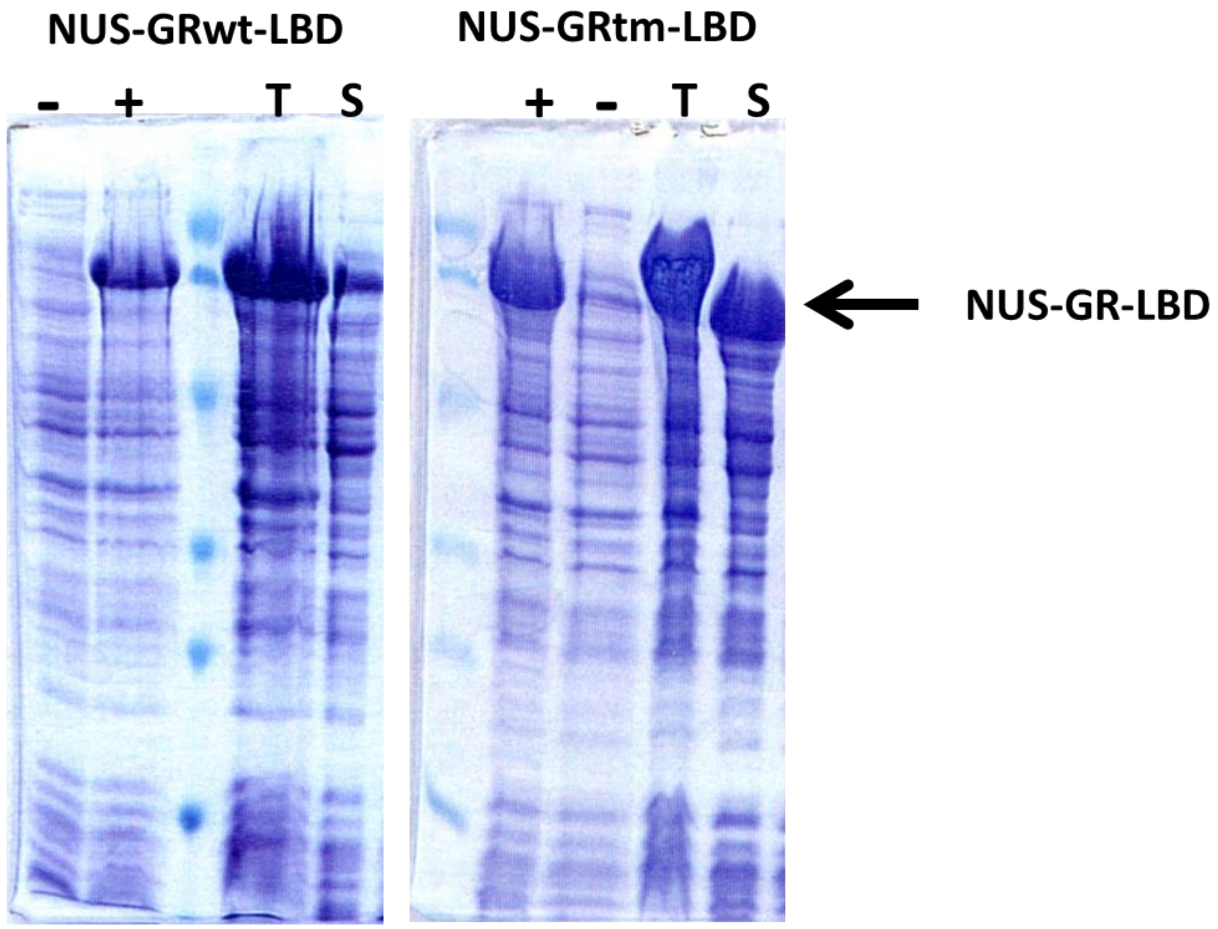

SDS PAGE on expression tests in *E. coli* for NUS-GRwt (524 – 777) and NUS-GRtm (524 – 777) with the mutations C636A, W557T and W712S. (-) before induction, (+) after induction, T: total extract, S: soluble extract. The NUS-GRtm construct enabled the production of increased amounts of soluble protein. Solubilization buffer: 250 mM NaCl, 50 mM Tris pH7.5, 10 μM dexamethasone.

Supplementary figure 11

TIF2	HIS		GST		MBP	
	LBS/18°C		LBS/18°C		LBS/18°C	
	T	S	T	S	T	S
378-828	**+**	**+/-**	**+**	**+**	**+++**	**++**
378-1008	nd	nd	nd	nd	**+++**	**++**
623-772	**+**	**+**	**+**	**+**	**+++**	**+++**
623-828	**+**	**+/-**	**+**	**+**	**+++**	**++**
623-1008	**+**	**+/-**	**+**	**+**	**+++**	**++**

Expression tests in *E. coli* for 5 constructs of TIF2 in fusion with HIS, GST and MBP. T: Total Extract, S: Soluble extract, LBS: LB medium for cell culture plus 10% sucrose. The sequence 623-772 yielded the highest amount of soluble protein. We chose the fusion with the small HIS tag for complex reconstitution with GR and ERβ. Solubilization buffer: 50mM Phosphate Na/K pH 7.5, 50 mM NaCl. The intensity of the band corresponding to the protein of interest was compared to the total amount of proteins. The gradation corresponds to the absence of expressed protein (-) up to 50% of the total amount of proteins (++++).

Supplementary figure 12

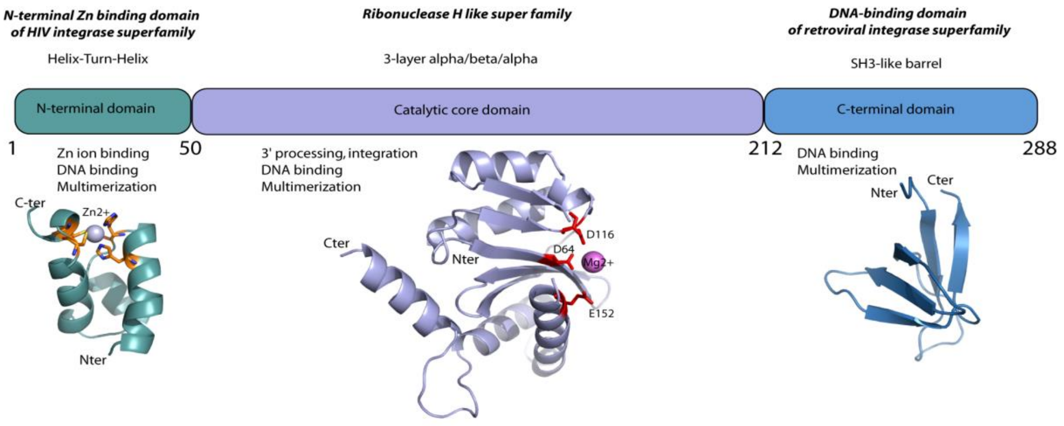

Domain organization and known domain structures (Cai et al., 1997, Dyda et al., 1994, Lodi et al., 1995) of HIV-1 integrase

Cai, M. et al. Solution structure of the N-terminal zinc binding domain of HIV-1 integrase. *Nat Struct Biol* **4**, 567-577 (1997).

Dyda, F. et al. Crystal structure of the catalytic domain of HIV-1 integrase: similarity to other polynucleotidyl transferases. *Science.* **266**, 1981-1986 (1994).

Lodi, P.J. et al. Solution structure of the DNA binding domain of HIV-1 integrase. *Biochemistry.* **34**, 9826-9833 (1995).

Supplementary figure 13

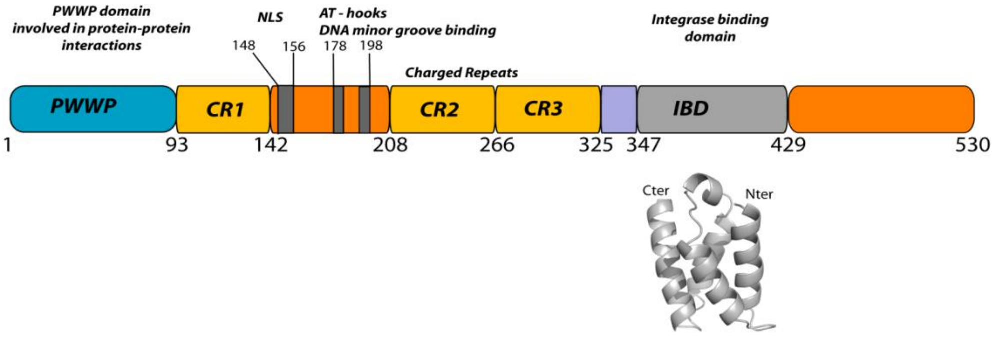

Domain organization and structure of the integrase binding domain (Cherepanov et al., 2005) of human LEDGF

Cherepanov, P., Ambrosio, A.L., Rahman, S., Ellenberger, T. & Engelman, A. Structural basis for the recognition between HIV-1 integrase and transcriptional coactivator p75. *Proc.Natl.Acad.Sci.U.S.A.* **102**, 17308-17313 (2005).

Supplementary figure 14

CDD Domains predictions

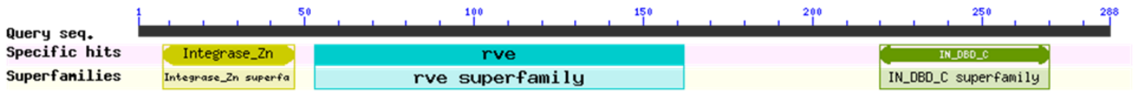

PONDR disorder analysis

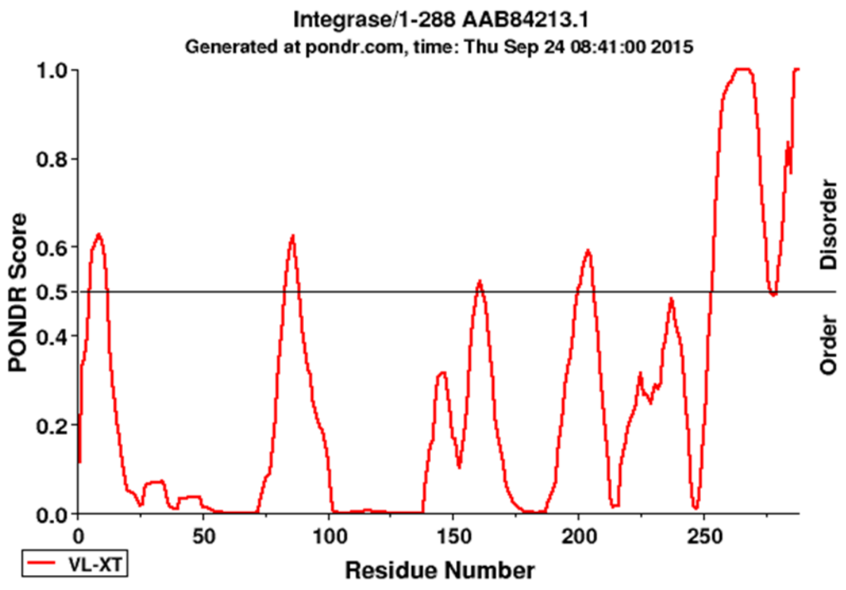

PROSO II and SOLPRO solubility predictions

Protein	PROSO II Predicted class	PROSO II Solubility score	SOLPRO Predicted solubility	SOLPRO Probability
HV-1 IN Full length	insoluble	0.545	soluble	0.956

IN domains prediction with CDD, disorder analysis with PONDR and solubility predictions with PROSO II and SOLPRO.

Supplementary figure 15

CDD Domains predictions

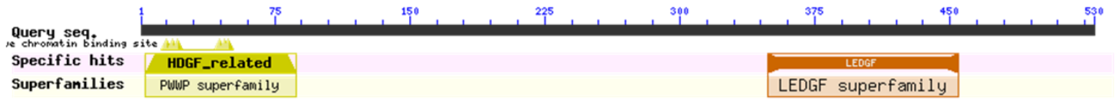

PONDR disorder analysis

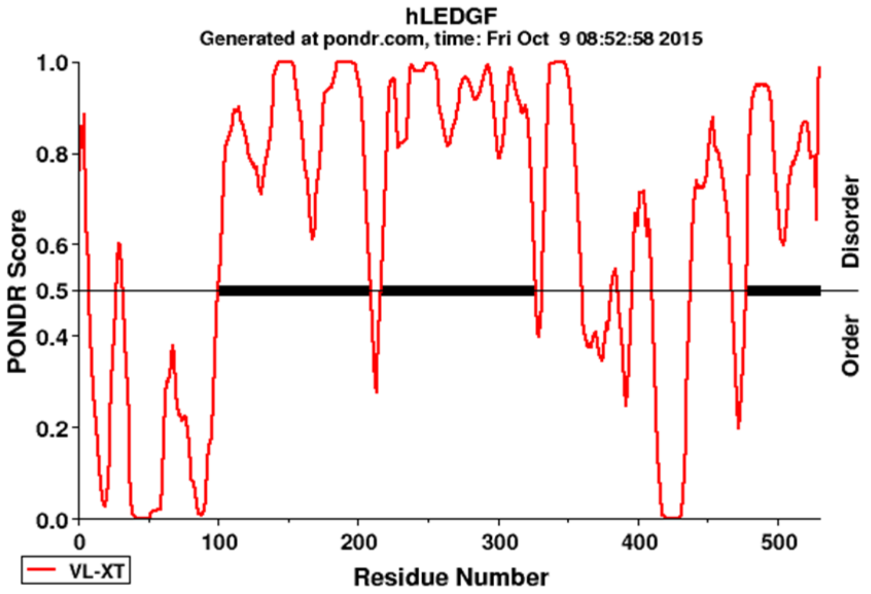

PROSO II and SOLPRO solubility predictions

Protein	PROSO II Predicted class	PROSO II Solubility score	SOLPRO Predicted solubility	SOLPRO Probability
hLEDGF Full length	soluble	0.729	soluble	0.821

LEDGF domains prediction with CDD, disorder analysis with PONDR and solubility predictions with PROSO II and SOLPRO

Supplementary figure 16

A (1)

	37°		25°		18°	
	ET	EB	ET	EB	ET	EB
LB	+	-	+	+	+	+
LB/sucrose	+	-	+	+	++	++

(2)

	-	Glycerol 10%	Chaps 10mM	Triton 0.1%	Sucrose 20%
50mM NaCl	+/-	+/-	++	+/-	+/-
1M NaCl	+	++	+++	+++	++

B (1) 1M NaCl + (2) 7mM CHAPS

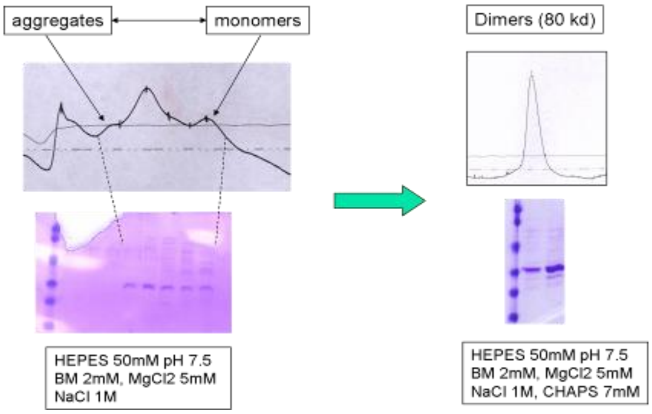

HIV-1 integrase solubility assay (E. coli expression). A1: The best conditions are overnight expression at 18°C with 10% sucrose in the LB cell culture medium. A2: The best solubility buffer is 1M NaCl with 10 mM CHAPS. ET: Total Extract, ES: Soluble extract. LB: LB medium for cell culture, LB/sucrose: LB medium for cell culture plus 10% sucrose. B: Gel filtration profile and SDS-PAGE on eluted fractions in 1M NaCl in absence (B1) and in presence (B2) of 7 mM CHAPS. Without CHAPS, HIV-1 IN is in aggregating whereas after adding 7 mM CHAPS, only dimers are present.

Supplementary figure 17

Protein	Fragment	Tag Nter	Protease	Antibiotic	Expression	Solubility
Integrase	1-288	-	No	Chlo	+	+
		-	Thrombin	Amp	+	+
		HIS		Amp	++++	+++
		HIS		Zeocin	++	++
		HIS		Kana	++	++
		GST		Amp	++	+++
		MBP		Amp	+++	+++
		HIS-NUS		Amp	++	+++
		HIS	P3C	Amp	++	+++
		GST		Amp	+++	+++
		HIS		Chlo	+	+

Expression and solubility assays for HIV-1 full length integrase (E. coli expression). All construct tested enabled protein expression and solubility. The GST IN was used for IN/LEDGF complex reconstitution. The optimized solubilization buffer contains 1M NaCl, 7mM CHAPS, HEPES 50mM pH=7.5, 5mM MgCl2, 2mM βmercaptoethanol (**Supplementary Fig. 16**). Chlo: chloramphenicol, Kana: kanamycin, Amp: ampicillin. The intensity of the band corresponding to the protein of interest was compared to the total amount of proteins. The gradation corresponds to the absence of expressed protein (-) up to 50% of the total amount of proteins (++++).

Supplementary figure 18

Protein	Fragment	Tag Nter	Protease	Antibiotic	Expression	Solubility
LEDGF	1-530	-	-	Chlo	++	++
		-	Thrombin	Amp	++	+++
		HIS		Amp	+++	+++
		HIS		Zeocin	+++	+++
		GST		Amp	+	+++
		MBP		Amp	+	+++
		HIS-NUS		Amp	+	+++
		HIS	TEV	Amp	+++	++
		GST		Amp	+	++
		HIS	P3C	Amp	+++	++
		GST		Amp	+	++
		HIS		Spec	+++	++
	347-429	HIS	P3C	Amp	+++	++
		GST		Amp	+++	+++
		MBP		Amp	+++	++

Expression and solubility assays for human LEDGF full length and for the integrase binding domain (347-429) (E. coli expression). All constructs tested enabled protein expression and solubility. The HIS LEDGF was used for IN/LEDGF complex reconstitution. Chlo: chloramphenicol, Kana: kanamycin, Amp: ampicillin. The same solubilization buffer as for HIV-1 IN was used (1M NaCl, 7mM CHAPS, HEPES 50mM pH=7.5, 5mM $MgCl_2$, 2mM βmercaptoethanol). The intensity of the band corresponding to the protein of interest was compared to the total amount of proteins. The gradation corresponds to the absence of expressed protein (-) up to 50% of the total amount of proteins (++++).
