# Supplementary figures and images for: Production of unstable proteins through the formation of stable core complexes
Source: Nat Commun. 2016 Mar 17;7:10932. doi: 10.1038/ncomms10932 (PMC4800440; doi:10.1038/ncomms10932)

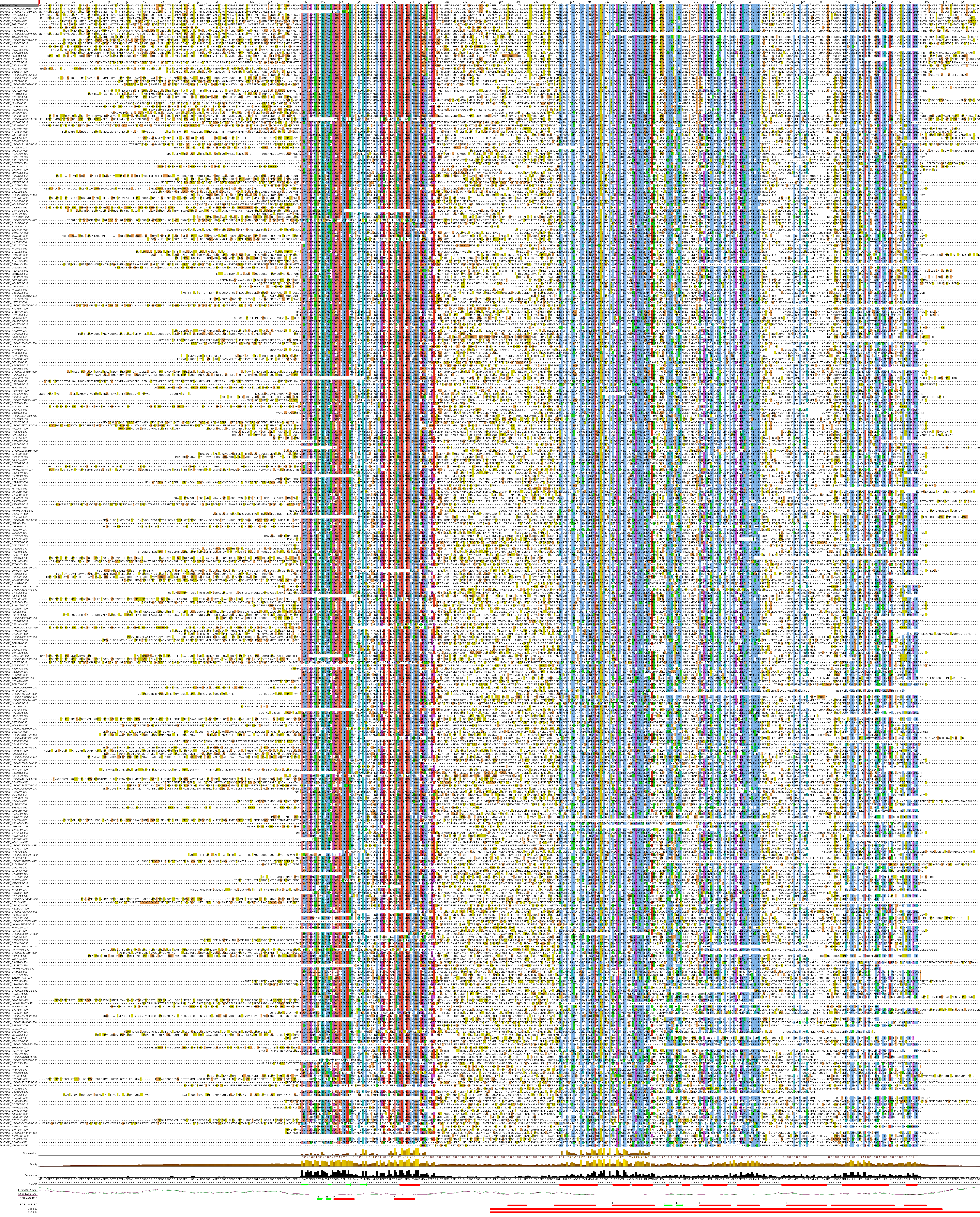

Supplement: Supplementary Data Set 1 — Human ERβ Sequence multi alignment and domains predictions [file ncomms10932-s2.png]

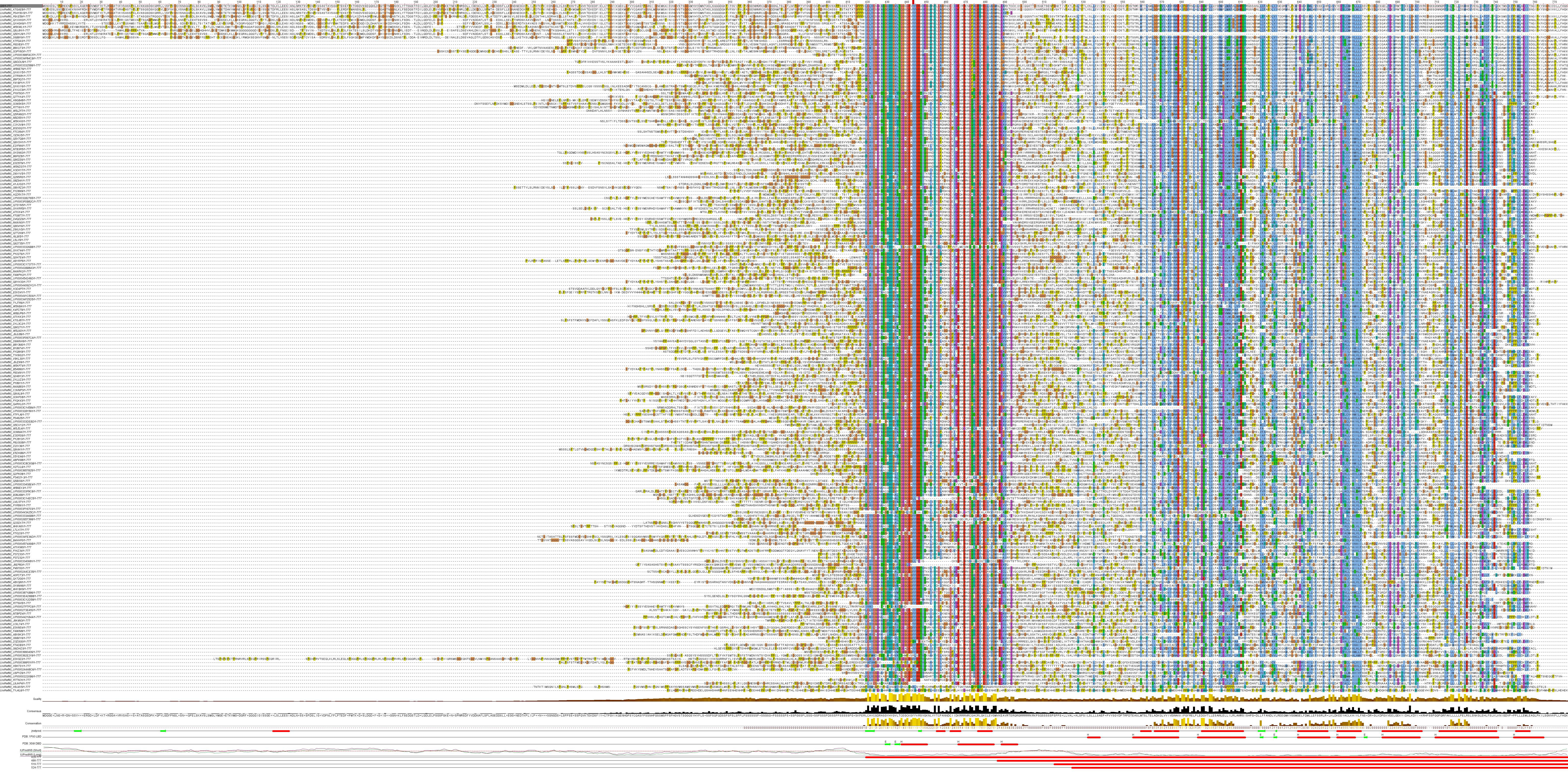

Supplement: Supplementary Data Set 2 — Human GR Sequence multi alignment and domains predictions [file ncomms10932-s3.png]

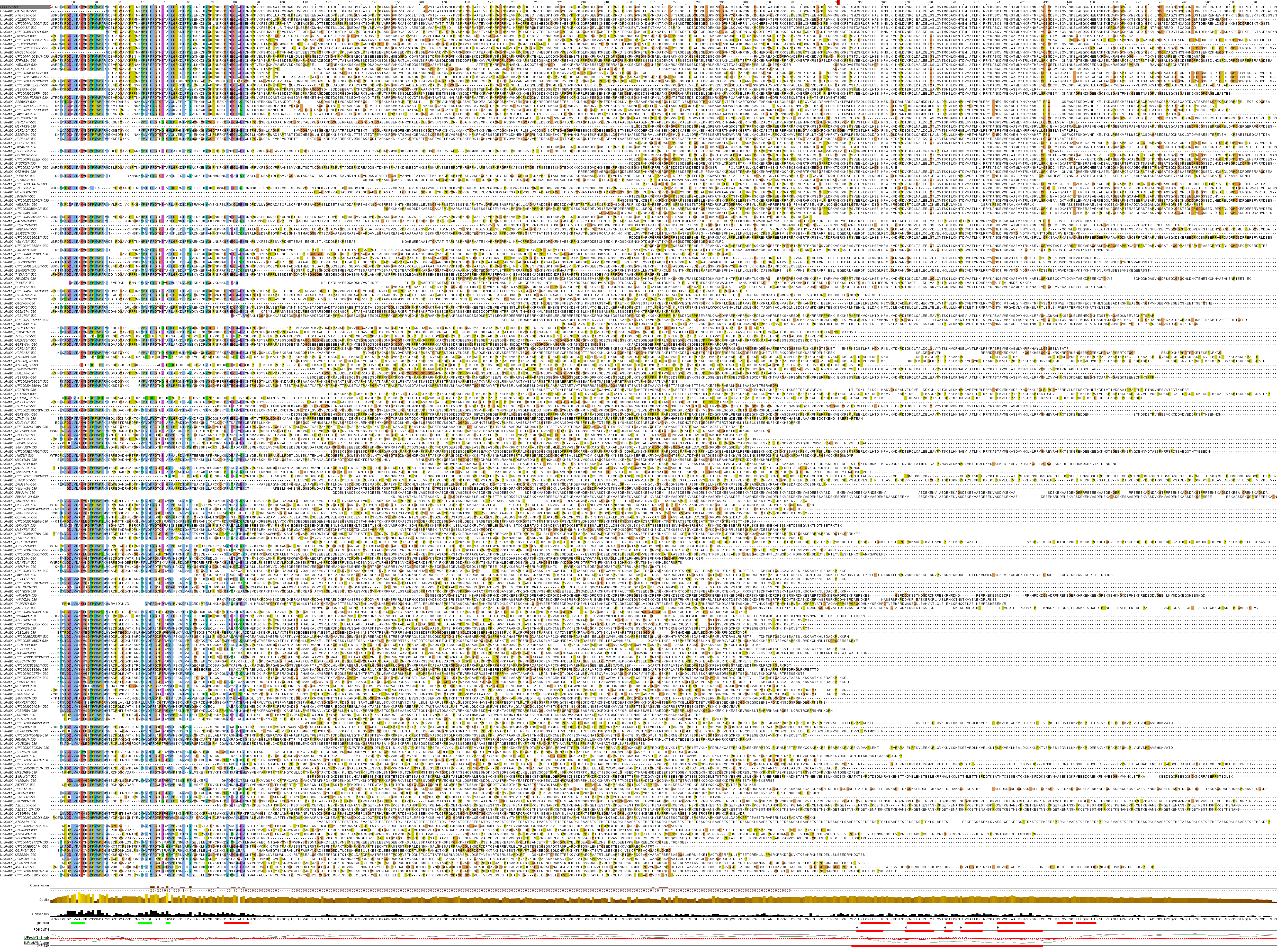

Supplement: Supplementary Data Set 5 — Human LEDGF Sequence multi alignment and domains predictions [file ncomms10932-s6.png]
